# Effect of the several epoxy resin-based sealer compositions on adhesion interface in radicular dentin after calcium hydroxide intracanal medication removal

**DOI:** 10.4317/jced.58375

**Published:** 2021-09-01

**Authors:** Victor-Feliz Pedrinha, Cristiane-de Melo Alencar, Fernanda-Ferreira-de Albuquerque Jassé, Joissi-Ferrari Zaniboni, Andréa-Abi-Rached Dantas, Flaviana-Bombarda de Andrade, Milton-Carlos Kuga

**Affiliations:** 1 Department of Restorative Dentistry, Endodontics and Dental Materials, Bauru School of Dentistry, University of São Paulo (USP), Bauru, SP, Brazil; 2 Department of Restorative Dentistry, Araraquara School of Dentistry, State University of São Paulo (UNESP), Araraquara, SP, Brazil; 3 School of Dentistry, Federal University of Pará (UFPA), Belém, PA, Brazil

## Abstract

**Background:**

This study evaluated the effects of several epoxy resin-based sealer compositions (AHP, AH Plus; ADS, Adseal; SPL, Sealer Plus) on bond strength and intratubular dentin penetration of the endodontic obturation, in root canal previously treated with calcium hydroxide intracanal medication (CH) and removed by continuous ultrasonic irrigation (CUI).

**Material and Methods:**

Forty-five maxillary canines were prepared up to F5 (ProTaper system), filled with CH, coronally sealed, and stored at 37ºC. After 1 week, CH was removed using 2.5% sodium hypochlorite energized by CUI. The specimens were randomly distributed in three groups (n=15) and root canal obturated, according to epoxy-based resin sealer composition (AHP, ADS or SPL). The roots were transversally sectioned in cervical, middle, and apical thirds. In each radicular third, push out bond strength using universal machine and intratubular dentin using confocal laser scanning microscopy (CLSM) and Image J Program were evaluated. Bond strength and intratubular dentin penetration were statistically evaluated by ANOVA one-way and Tukey tests and Kruskal Wallis test, respectively (α = 0.05).

**Results:**

In middle and apical thirds, AHP showed higher bond strength values (*p*<0.05), and ADS and SPL were similar each other (*p*>0.05). All epoxy resin-based sealers presented similar intratubular dentin penetration, independently of the radicular thirds (*p*>0.05). Cohesive and mixed failures were predominant in the cervical thirds. In the middle and apical thirds, AHP showed more cohesive type failures, while ADS and SPL showed more adhesive-type failures.

**Conclusions:**

AHP has the highest bond strength in middle and apical radicular thirds, after calcium hydroxide intracanal medication removal using continuous ultrasonic irrigation, although intratubular dentin infiltration being similar among epoxy resin-based sealer with several chemical composition.

** Key words:**Continuous ultrasonic irrigation, endodontic sealers, epoxy resin-based sealers, root canal obturation.

## Introduction

The hermetic sealing of the root canals after chemical-mechanical preparation consists in a crucial step for the long-term success of endodontic therapy, preventing bacterial leakage and development of apical periodontitis (1,2). Since gutta-percha is not able to adhere to dentinal walls, endodontic sealers are used to fill the irregularities and smaller dentinal tubules of the root canals (2). Although several new endodontic sealers are commercially available, none had all the physicochemical and biological properties recommended to be selected as the ideal material for root canal obturation, especially in relation to dentin substrate adhesion. (3,4). However, epoxy resin-based sealers are the most used in current clinical practice, owing to their satisfactory properties (5), including high push-out bond strength and lower solubility (6,7).

AH Plus (AHP; Dentsply DeTrey, Konstanz, Germany) is an epoxy resin-based sealer used frequently as a gold standard for comparison with new endodontic sealers, due to its good physicochemical properties and provide satisfactory adhesion interface with dentin radicular surface (1-5). Recently, two new epoxy resin-based sealer were introduced in endodontic arsenal, but with different chemical compositions (1,2,8). Adseal (ADS; Meta Biomed, Cheongju, South Korea) has calcium phosphate, calcium oxide and salicylate resin in its chemical composition (1,2). On the other hand, Sealer Plus (MK Life, Porto Alegre, RS, Brazil) has calcium hydroxide, hexamethylenotetramine and bisphenol resin (8,9). Although these endodontic sealers have epoxy resin in their composition, they demonstrate different chemical results and biological compatibility (1,2,8,9). However, there are no comparative studies regarding the bond strength and dentinal penetrability of these epoxy resin-based sealers on the root canal dentin.

In some clinical situations, it is impossible to complete root canal treatment in the same session (3,10,11). In these cases, is recommended the use of calcium hydroxide (CH) intracanal medication (10,11). However, CH should be completely removed from the root canals before endodontic obturation because CH residues can negatively interfere on adhesion interface with radicular dentin and/or physical properties of the epoxy resin-based endodontic sealer (12). An alternative to solution this problem is continuous replenishment of irrigation solutions, such as continuous ultrasonic irrigation (CUI).

Therefore, the objective this study was to evaluate the effects of several epoxy resin-based sealer compositions (AHP, AH Plus; ADS, Adseal; SPL, Sealer Plus) on bond strength and intratubular dentin of the endodontic obturation, in root canal previously treated with calcium hydroxide intracanal medication (CH) and removed by continuous ultrasonic irrigation (CUI), in cervical, middle, and apical root canal thirds. Similar bond strength and intratubular dentin penetration, independently of the root canal thirds, were considered as the null hypothesis H01 e H02, respectively.

## Material and Methods

-Sample Preparation

This study followed the ethics recommendations for human research and was approved by the Local Research Ethics Committee (CAAE: 20822914.2.0000.5416). Previous studies were used to determine the sample size (13,14). Forty-five human maxillary canines, recently extracted due to periodontal diseases, were selected. Mesiodistal and buccolingual periapical radiographs were recorded to select only teeth with similar characteristics as follows: a single-root and a single straight canal with fully formed apices, and absence of calcifications or endodontic treatment. The selected teeth were stored in 0.1% thymol solution at 4ºC before the experimental tests.

A single operator performed all the experimental steps. Teeth were decoronated with a diamond disc (KG Sorensen, São Paulo, SP, Brazil) under copious irrigation, and the roots were standardized to 16 mm length. The canals were explored using #15 K-files (Dentsply Maillefer, Ballaigues, Switzerland) until the tip was juxtaposed to the apical foramen. The working length (WL) was defined as being 1 mm shorter than the total canal length. The apical foramens were sealed with composite resin. Root canal were enlarged up to F5 instrument (ProTaper System; Dentsply Maillefer, Ballaigues, Switzerland) to standardize their diameters. The enlargement of the canals was performed according to the manufacturer’s recommendations and irrigated with 5 mL of 2.5% sodium hypochlorite (NaOCl) solution through a 30-gauge needle (NaviTip; Ultradent, South Jordan, UT) before each instrument change. Next, 5 mL of 17% ethylenediaminetetraacetic acid (EDTA) was used for 3 min, followed by 5 mL of 2.5% NaOCl. The canals were rinsed with 10 mL distilled water and dried with F5 absorbent paper points (ProTaper, Dentsply Maillefer, Ballaigues, Switzerland).

The UltraCal XS (Ultradent, Salt Lake, UT, USA) paste was delivered into the root canals up to the established WL, and the filling quality was checked radiographically. The access cavities were sealed with glass-ionomer cement (Maxxion R; FGM, Joinville, SC, Brazil), and the specimens were stored at 37ºC and 100% humidity for 1 week. Temporary filling materials were removed, and #15 K-file (Dentsply Maillefer, Ballaigues, Switzerland) was introduced to loosen the paste and create space for the irrigation needle.

The root canals were irrigated with 50 mL of 2.5% NaOCl simultaneously with continuous ultrasonic irrigation (CUI) using an ultrasonic tip (Irrisonic E1; Helse, Santa Rosa de Viterbo, SP, Brazil), 2 mm short of the WL, adapted in ultrasonic device (Various II; NSK Dental, Tokyo, Japan), by 1 minute. The procedure was conducted with vertical movements in the apico-cervical directions. In sequence, the root canals were aspired, followed by conventional syringe irrigation using a 30-gauge needle (NaviTip; Ultradent, South Jordan, UT, USA), with 5 mL of 17% EDTA for 3 min, 5 mL of 2.5% NaOCl, and 10 mL of distilled water. Then, the root canals were dried with absorbent paper points.

Table 1 shows the identification, compositions and manufacturer information of the epoxy resin-based sealers evaluated in this study. Endodontic sealers were used according to the manufacturer’s instructions, in a 1:1 proportion of the base and catalyst pastes. To provide fluorescence in the confocal laser scanning microscopy (CLSM) assessment, the sealers were mixed with 0.01% Rhodamine B dye (Sigma-Aldrich, St. Louis, MO, USA), in weight. The roots were randomly distributed into three groups (n=15, each), according to chemical epoxy-epoxy resin sealer: AHP, ADS and SPL groups. The sealers were inserted into the canals, 1 mm short of the WL, using a 600-rpm Lentulo spiral (Dentsply Maillefer, Ballaigues, Switzerland), adapted in electric endodontic motor (X-Smart Plus, Dentsply Maillefer, in 2N (3).

**Table 1:**
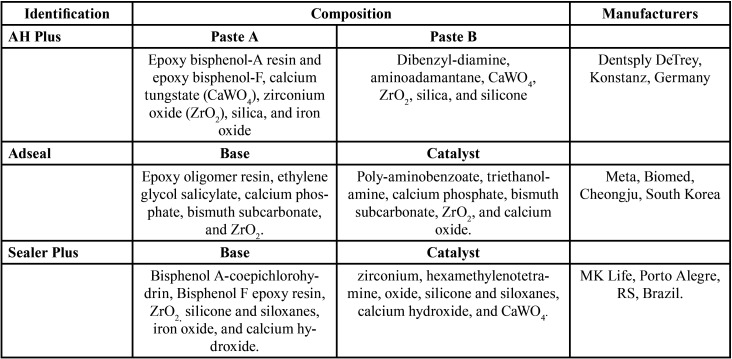
Identification, composition and manufacturer informations of the epoxy resin-based sealer.

Next, the sealers were activated by E1 tip for 20 s, at a mode 1 of the power setting, and an F5 gutta-percha cone (ProTaper; Dentsply Maillefer, Ballaigues, Switzerland) was introduced into the canal. The cervical excess of gutta-percha was removed using a flame-heated plugger, and cold vertical condensation was performed. The root canal orifices were sealed temporarily with glass–ionomer cement (Maxxion R; FGM, Joinville, SC, Brasil), stored in 37ºC, in 100% humidity, for 1 week. In this way, the sealers were able to set completely. Radiographs were taken in different directions to check the obturation quality.

-Push-out Bond Strength analysis

The roots were vertically placed inside a plastic matrix and included in polyester resin (Maxi Rubber, Diademam SP, BR), up to 15 mm, leaving 1 mm of the cervical third outside inclusion. After 24 hours, the specimens were removed from the plastic matrices and each root was transversally sectioned using a water-cooled low-speed diamond-coated disk (Isomet 2000, Buehler Ltd, Lake Bluff, IL, USA) (11,12). Three slices with 2 mm (±1 mm) thickness were prepared from the cervical, middle, and apical thirds of each root located 1 mm, 5 mm, and 10 mm, respectively, from the cervical line. Irregularities were removed using #1200 sandpaper (Norton, São Paulo, SP, Brazil), followed by cleaning with brush and air jets.

The push-out test involved applying a constant compressive load using an electromechanical testing machine (EMIC, DL2000, São José dos Pinhais, PR, Brazil), at 0.5 mm/min in the apical-coronal direction until failure, using a 5-kN cell. A cylindrical crosshead of 1.3 mm, 0.9 mm, and 0.5 mm diameters, respectively in the cervical, medial, and apical thirds, was positioned to provide the displacement of the root canal obturation. The values were initially obtained in “Newtons” and posteriorly converted into tension (MPa) (14,15).

-Failure mode analysis

After the push out bond strength test, all specimens were analyzed under a stereomicroscope (Leica Microsystems, Wetzlar, Germany), at ×20 magnification. The failure patterns were classified as adhesive type-1 (failure at the sealer-core interface), adhesive type-2 (failure at the sealer-dentin interface), mixed (both adhesive and cohesive failures), and cohesive (failure within the sealer) (16).

-Intratubular dentin penetration analysis

Slices corresponding to each radicular third were visualized by CLSM using a Leica TCS-SPE confocal microscope (Leica Microsystems GmbH, Mannheim, Germany), at 100x magnification, to determine the root canal perimeter with material penetration within the dentinal tubules. Images were acquired using the Leica Application Suite-Advanced Fluorescence (Leica Mannheim, Germany) software. The penetration area of endodontic sealer in the radicular dentin was measured as previously described (14).

-Statistical Analysis

The homoscedasticity data were initially verified by Shapiro-Wilk test. The penetration data were analyzed by Kruskal-Wallis and Dunn post-hoc tests. Since bond strength presented normality, it was evaluated using one-way analysis of variance and Tukey tests (α = 0.05).

## Results

-Push-out Bond Strength analysis

In cervical root canal third, all push out bond strength values were similar, independently of the epoxy resin-based sealer composition (*p* > 0.05). In middle and apical root canal thirds, AHP showed the highest push out bond strength value (*p* < 0.05), while ADS and SPL showed similar values (*p* > 0.05). Table 2 shows the means and standard deviations (in MPa) of the bond strength of the several epoxy resin-based compositions sealers, after calcium hydroxide removal using continuous ultrasonic irrigation, in different root canal thirds.

**Table 2:**
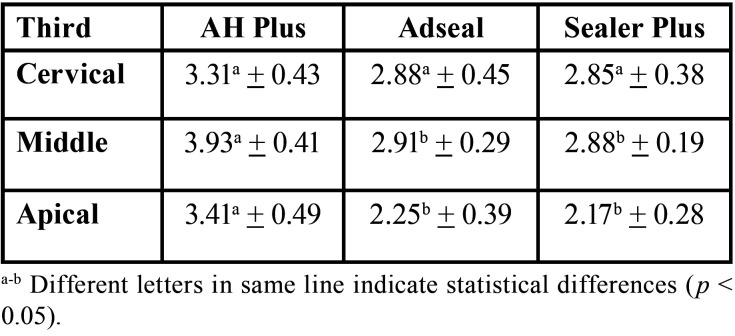
Mean and standard deviations (in MPa) of push out bond strength of epoxy resin-based endodontic sealers after intracanal calcium hydroxide removal, in each root canal third.

In the cervical third, cohesive and mixed failures were most common for all sealers. In the middle and apical thirds, AHP showed more cohesive-type failures, whereas ADS and SPL showed more adhesive-type failures.

-Failure mode analysis

**Figure 1:**
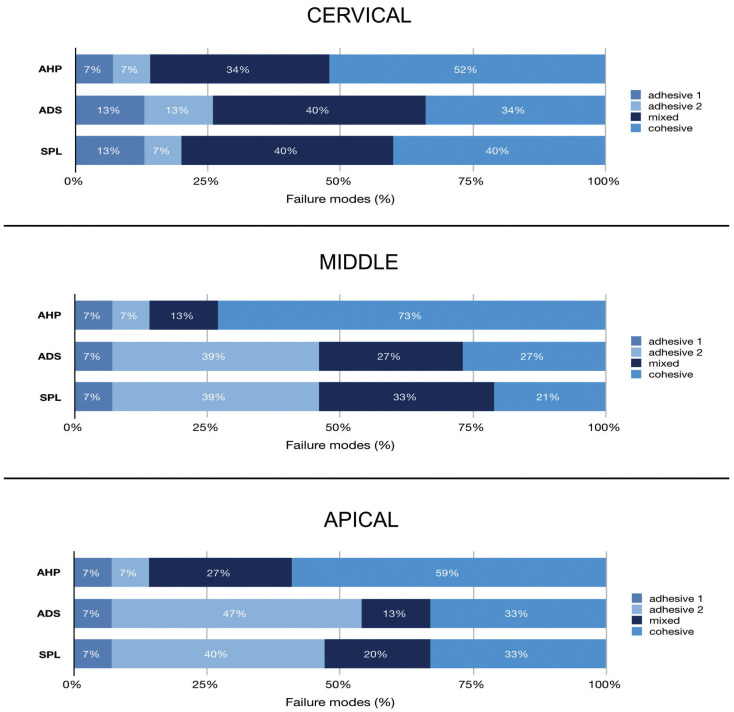
Distribution of the failure modes after the push out bond strength analysis to AH Plus (AHP), Adseal (ADS), and Sealer Plus (SPL) groups.

**Figure 2:**
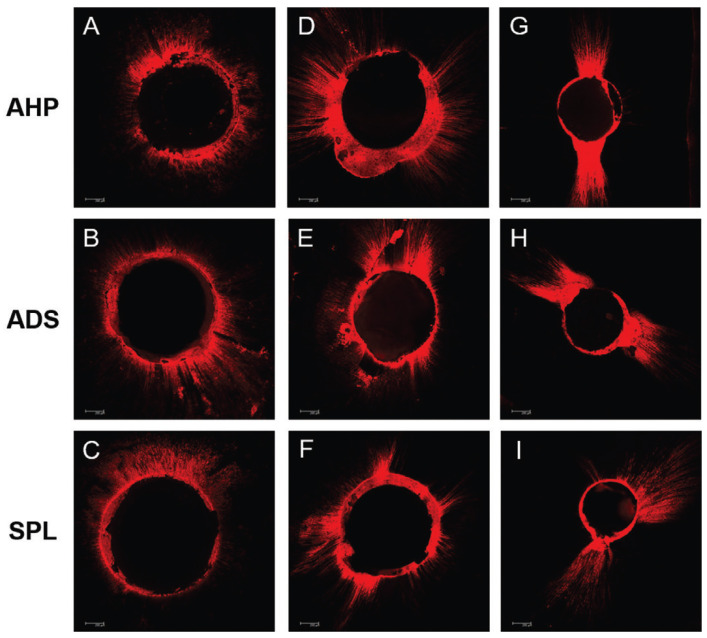
Representative images of the intratubular dentin penetration to epoxy resin-based sealers: (A-C): cervical third of the roots; (D-F): middle third of the roots; (G-I) apical thirds of the roots (AHP: AH Plus; ADS: Adseal; SPL: Sealer Plus) (Rhodamine B dye, 100x).

Failure mode was expressed as frequency in function of the epoxy resin-based sealer composition and root canal third. In cervical third, cohesive and mixed failures were most common for all sealers. In the middle and apical thirds, AHP showed more cohesive-type failures, whereas ADS and SPL showed more adhesive-type failures (Figs. 1,2).

Figure 1 shows the incidence (in percentage) of the failure mode of the several epoxy resin-based composition sealers, after calcium hydroxide removal using continuous ultrasonic irrigation, in different root canal thirds.

-Intratubular dentin penetration analysis

All epoxy resin-based sealers presented similar intratubular penetration, independently of the chemical composition or root canal thirds (*p* > 0.05). Table 3 shows the median, minimum, and maximum values (in %) of the dentin intratubular penetration of the several epoxy resin-based compositions sealers, after calcium hydroxide removal using continuous ultrasonic irrigation, in different root canal thirds.

**Table 3:**
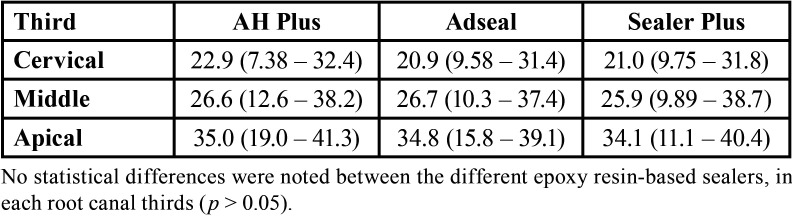
Median, minimum, and maximum values (%) of intratubular dentin penetration of the epoxy resin-based sealers, according to root canal thirds.

## Discussion

In the present study, after CH intracanal medication removal by CUI, the AHP sealer showed the highest bond strength in middle and apical root canal thirds. However, all epoxy resin-based sealers presented similar intratubular dentin penetration, independently of the radicular thirds. Therefore, H01 was rejected and H02 was accepted.

Push out bond strength and dentin intratubular penetration are methods routinely used to evaluate the adhesion between endodontic sealers and root canal dentin (2-4,17,18). However, push out bond strength evaluation has been applied in root canal obturation with or without gutta percha (19). In our study, in order to perform it similar to clinical conditions, the root canal was obturated using gutta percha and epoxy resin-based sealer, and maximum contact area between the tip crosshead and root canal obturation was observed (19). On the other hand, since the tips crosshead and root canal had different diameters, push out bond strength evaluation were performed only in the same radicular third between endodontic sealers (15).

In our push out analysis, another care taken was to apply debonding force axially in apico-cervical direction, avoiding stress on the surrounding canal walls (14,17). Thus, the stress distribution was uniform, enabling measurements to demonstrate the real interfacial bond strength between root canal dentin and epoxy resin-based sealers (20,21).

Endodontic sealers should provide adhesion between the gutta-percha and root canal walls, avoiding occurrence of gaps at the sealer-dentin interface (2,22). In our study, AHP showed better bond strength in the middle and apical thirds (*p* < 0.05), while ADS and SPL were similar each other (*p* > 0.05). Crucially, in the middle and apical root thirds, AHP showed higher cohesive incidence failure mode, whereas ADS and SPL demonstrated a higher adhesive type-2 failure mode.

It is possible that the different chemical composition of epoxy resin-based sealer may have effects on push out bond strength values. Previous studies showed higher bond strength with AHP, corroborating our findings (3,18). The AHP sealer is available in two pastes, A (base) and B (catalyst), containing calcium tungstate, zirconium oxide, and silica in both pastes. Paste A additionally contains the epoxy resins bisphenol A and F and iron oxide, while paste B contains silicone oil, dibenzyl-diamine, and aminoadamantane. On the other hand, these last two chemical components are not found in ADS and SPL sealers. The miscibility between the organic (resins) and inorganic (other substances) components of the two pastes also could be responsible for these results (23).

Another relevant clinical aspect addressed in our study was the previous use of the CH paste as intracanal medication. Although some studies show that previous CH medication does not affect the bond strength of AHP (24,25), there is consensus that all intracanal medication must be removed before root canals obturation (26), but CH residues may remain in apical root canal third (27). Considering the hypotheses established, the present investigation includes only the performance of different epoxy resin-based sealer compositions from a situation that CH was used as intracanal medication. In view of this, a comparison in conditions without intracanal medication was not performed.

Moreover, CH residues on the root canal walls negatively affects the endodontic sealers intratubular penetration (12,26). In our study, CUI was used as intracanal medication removal protocol. This irrigation system was chosen due to it provides continuous solution delivery and simultaneous ultrasonic activation. NaOCl activated by CUI protocol improves tissue dissolution, due to its continuous release of oxygen singlet and chlorine (28). As CUI protocol was performed 2 mm short of the WL, probably CH residues persisted in apical and middle root canal thirds (26,27), that interfered mainly on bond strength SPL and ADS groups. This topic can be linked with the common occurrence of cohesive and mixed failures in the cervical root third. Probably, in the cervical third is where the greatest removal of CH occurs due to the irrigation effects. So, the sealers penetrability can favor the adhesion to dentine walls in this area. Therefore, adhesive failures were commonly found in apical root thirds, especially in ADS and SPL group.

However, it was not enough to affect the epoxy resin-based sealers intratubular penetration. These results can be attributed to the ultrasonic activation of the endodontic sealers, because the heat generated during activation reduces the sealer viscosity, provides better flow, and incorporates sealer particles into the dentin organic matrix (3,26,29). Therefore, ultrasonic activation favored greater dentinal sealer penetration (1).

Our results corroborated with previous findings, where SPL presented lower bond strength when compared to AHP, in middle and apical root canal thirds (3). The possible explanation for these founds is due to CH residues reacting with phosphate ions of the dentin substrate resulting in a chemical smear layer that negatively interferes on bond strength of some endodontic cements (3,27-32).

Our results were obtained in laboratorial conditions, where push out bond strength and intratubular dentin penetration were evaluated. Every *in vitro* study must be carefully interpreted, and other complementary results must be obtained, especially the verification that the clinical evidence agrees with the laboratory findings. Despite these results, further studies assessing microbial infiltration and microscopic analysis of the interface adhesive between root canal dentin and endodontic obturation, under same clinical conditions as our study, should be evaluated.

## Conclusions

AHP has the highest bond strength in middle and apical radicular thirds, after calcium hydroxide intracanal medication removal using continuous ultrasonic irrigation, although intratubular dentin infiltration being similar among epoxy resin-based sealer with several chemical composition.
